# Extending a Standard SD-OCT Device to Ocular Biometry with Additional Software: Agreement and Clinical Applicability Compared with Ultrasound and Optical Biometry

**DOI:** 10.3390/jcm15218673

**Published:** 2026-11-06

**Authors:** İrfan Botan Güneş

**Affiliations:** Department of Ophthalmology Medar Hospital, Kocaeli Health and Technology University, 41275 Kocaeli, Türkiye; irfan.gunes@kocaelisaglik.edu.tr

**Keywords:** axial length, biometry, cataract, optical coherence tomography, ultrasonography

## Abstract

**Background/Objectives:** Although commercially available spectral-domain optical coherence tomography (SD-OCT) platforms are primarily designed for retinal and anterior segment imaging, ocular biometry can be enabled through additional software. This study evaluated the clinical applicability, interdevice agreement, and measurement success of this underexplored practical approach compared with conventional ultrasound and optical biometry. **Methods:** Clinical and biometric data of participants evaluated for refractive lens extraction (RLE) or cataract surgery were reviewed, and participants were classified into two groups according to lens opacity. Anterior chamber depth (ACD) and axial length (AL) measurements obtained with SD-OCT biometry (REVO 60, Optopol Technology, Zawiercie, Poland), ultrasound biometry (Accutome Inc., Malvern, PA, USA), and optical biometry (SW-9000 Plus, Suowei Electronic Technology, Tianjin, China) were analyzed. Measurement success and biometric measurements were evaluated separately within each group. Interdevice agreement was analyzed using intraclass correlation coefficients (ICCs) and Bland–Altman analysis. **Results:** This study included 207 eyes of 207 patients (62 RLE and 145 cataract eyes). Measurement success in the RLE group was 100% for all three methods. In the cataract group, success rates were 48.3% for SD-OCT, 80.0% for optical biometry, and 100% for ultrasound biometry. For AL, SD-OCT showed excellent agreement with ultrasound and optical biometry in both the RLE (ICC = 0.978 and 0.980, respectively) and cataract groups (ICC = 0.981 and 0.984, respectively). For ACD, agreement with ultrasound and optical biometry was good in the RLE group (ICC = 0.799 and 0.822, respectively) and moderate in the cataract group (ICC = 0.653 and 0.702, respectively). In the RLE group, AL measurements obtained with SD-OCT were significantly higher than those obtained with both ultrasound (*p* < 0.001) and optical biometry (*p* = 0.003). In cataract eyes, AL measurements did not differ significantly from ultrasound biometry (*p* = 0.066) or optical biometry (*p* = 0.859). **Conclusions:** SD-OCT biometry showed excellent agreement with established methods for AL when measurements were successfully obtained. However, measurement success in cataractous eyes was substantially lower than in optical biometry, limiting its current use as a standalone biometry method.

## 1. Introduction

Ocular biometry measures key ocular parameters, including axial length (AL), anterior chamber depth (ACD), corneal curvature, lens thickness (LT), and white-to-white distance (WTW), which can be used to calculate intraocular lens (IOL) power. Accurate biometric measurements are essential for achieving the intended refractive outcome after lens surgery. Among these parameters, AL is particularly important because errors in its measurement can substantially affect refractive prediction [1].

Several techniques are available for measuring AL. Ultrasound biometry has long been used for this purpose and remains valuable when optical acquisition is not possible. Optical biometry, however, has become the preferred noncontact method for cataract and refractive surgery because of its high precision and reproducibility [2,3,4].

Optical biometry initially relied on partial-coherence interferometry and optical low-coherence reflectometry, followed by the introduction of swept-source OCT (SS-OCT) systems. Modern SS-OCT biometers provide high repeatability and improved measurement success through lens opacities, although acquisition failure may still occur in advanced cataracts [5,6,7]. Spectral-domain OCT (SD-OCT) platforms are widely used for posterior and anterior segment imaging and are primarily designed for ocular imaging rather than biometry. Compared with SS-OCT, their shorter wavelength provides less penetration through opaque media because of greater light scattering and signal attenuation.

SD-OCT biometry is a software-based method for AL measurement using a commercially available SD-OCT platform, although clinical evidence regarding its use remains limited. Sikorski et al. [8] first described this method and reported high repeatability and agreement with a dedicated SS-OCT biometer. Zarei-Ghanavati et al. [9] later evaluated the method in cataractous eyes and also reported good agreement with optical biometers. However, previous studies primarily compared SD-OCT biometry with other optical methods, leaving limited evidence regarding its performance relative to a method independent of optical media transparency. Including ultrasound biometry provides an independent comparator, particularly when one optical device successfully obtains a measurement while the other fails. This approach allows a more comprehensive evaluation of measurement agreement and clinical applicability across different degrees of lens opacity.

Integrating biometry into an SD-OCT platform may increase its clinical utility by combining ocular imaging and biometric assessment within a single system, potentially reducing the need for a separate dedicated biometer.

The purpose of this study was to evaluate SD-OCT biometry by comparing AL and ACD measurements with those obtained using optical and contact ultrasound biometry. Interdevice agreement and measurement success were assessed separately in eyes with minimal lens opacity undergoing refractive lens extraction (RLE) and in cataractous eyes, allowing the clinical applicability of this method to be evaluated under different degrees of lens opacity.

## 2. Materials and Methods

This retrospective cross-sectional clinical study was conducted at Kocaeli Health and Technology University, Medar Hospital, following approval from the Institutional Ethics Committee of Kocaeli Health and Technology University (No.: 2026-33/21). Written informed consent was obtained from all participants before enrollment, and this study was conducted in accordance with the principles of the Declaration of Helsinki.

Medical records of consecutive patients who underwent biometric evaluation for refractive lens extraction or cataract surgery between November 2024 and July 2026 were reviewed, and one eye of each patient was included in the analysis. In participants with both eyes meeting the eligibility criteria, one eye was randomly selected for inclusion in this study. The refractive lens extraction group included 62 eyes with minimal lens opacity, whereas the cataract group included 145 eyes with clinically significant lens opacity. The two groups represented different clinical conditions and degrees of lens opacity and were therefore not directly compared. Instead, interdevice differences, agreement, and measurement success were evaluated separately within each group.

All patients underwent best-corrected visual acuity (BCVA) assessment, slit-lamp biomicroscopy, dilated fundus examination, and cataract grading according to the Lens Opacities Classification System III (LOCS III) [10]. BCVA was recorded as a baseline clinical characteristic. Cataract grading was performed by the same ophthalmologist, who was masked to the biometric measurement results.

Patients who underwent biometric evaluation for refractive lens extraction or cataract surgery between November 2024 and July 2026 were eligible for inclusion. The refractive lens extraction group comprised eyes with minimal lens opacity, while the cataract group included eyes with clinically significant nuclear cataract. Exclusion criteria were previous intraocular surgery, posterior subcapsular cataract, corneal pathology or opacity interfering with measurements, retinal pathology affecting fixation or measurement acquisition, and inability to cooperate with the examination. For SD-OCT biometry, scans with an image quality score below 6/10, the manufacturer-defined minimum acceptable threshold, were excluded.

Biometric measurements were obtained using three different techniques: OCT biometry (Revo 60, Optopol Technology, Zawiercie, Poland), contact ultrasound biometry (Accutome A-Scan Plus Connect 24-4400, Accutome Inc., Malvern, PA, USA), and optical biometry (Suoer SW-9000 Plus, Suowei Electronic Technology Co., Tianjin, China). All biometric measurements were performed under identical examination conditions by the same ophthalmologist, who was masked to the results obtained with the other devices at the time of each examination.

**Figure 1:**
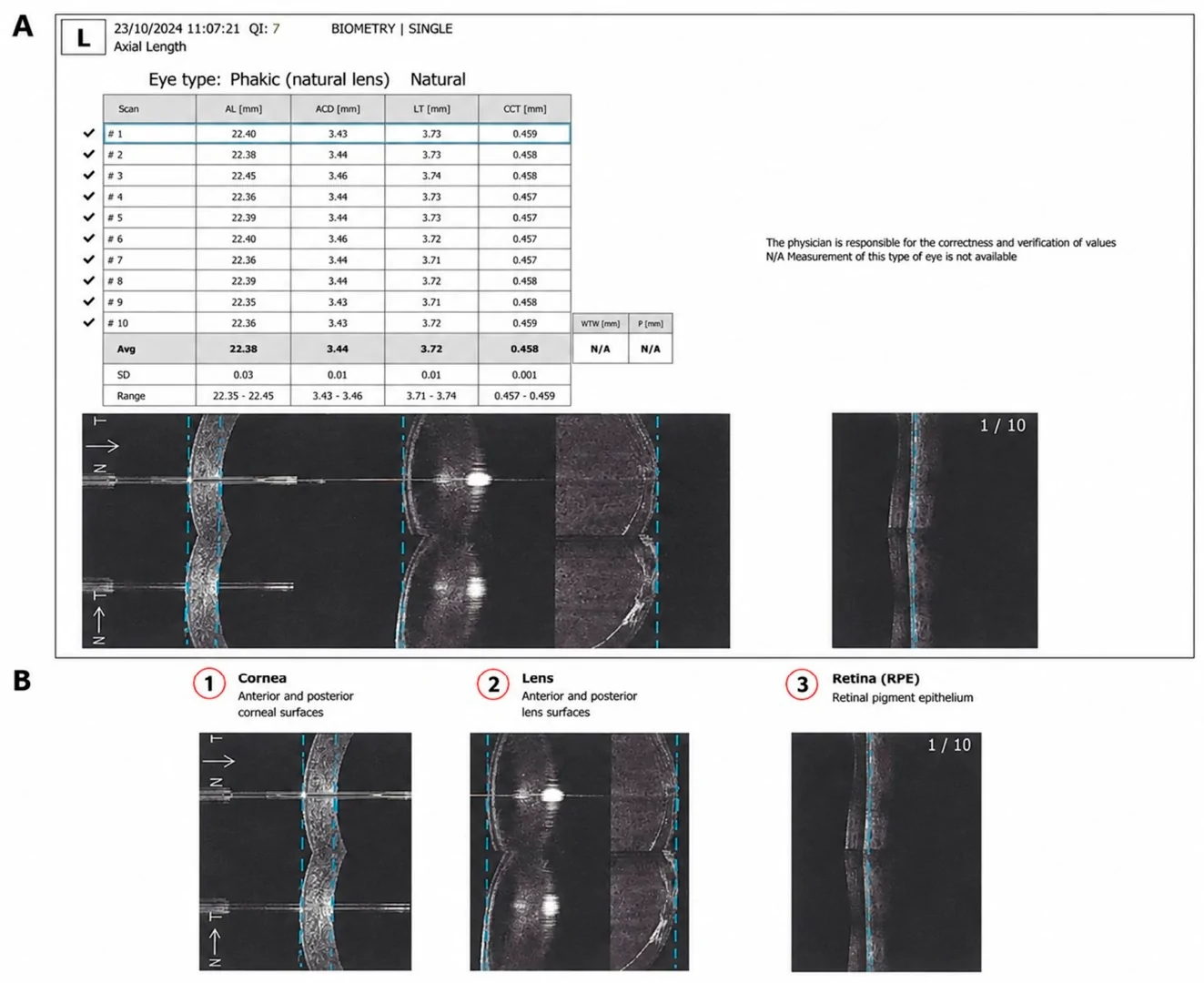
AL and ACD measurement using the spectral–domain OCT platform. (**A**) Representative integrated biometry report generated by the SD–OCT system. (**B**) Sequential OCT measurement windows used for biometric acquisition, including the cornea, crystalline lens, and retina/retinal pigment epithelium. Automated segmentation of the relevant anatomical interfaces is used to calculate anterior chamber depth and axial length.

SD-OCT biometry was performed using the manufacturer’s commercially available B-OCT biometry module integrated into the REVO 60 SD-OCT system, originally designed for anterior and posterior segment imaging, running SOCT software version 11.5.3. The system uses a superluminescent diode with a central wavelength of 850 nm and operates at 60,000 A-scans per second, with an axial resolution of approximately 5 µm in tissue. Unlike dedicated optical biometers, the system does not acquire the entire ocular axis in a single scan. Instead, biometric measurements are derived from four consecutive acquisition windows covering the cornea, crystalline lens, and retina. The software automatically identifies the relevant anatomical interfaces and combines the measurements to calculate AL, from the corneal apex to the retinal pigment epithelium at the fovea, and ACD, from the anterior corneal surface to the anterior lens surface. No manual segmentation corrections were performed. The measurement principle is illustrated in Figure 1. For each examination, 10 consecutive measurements were automatically acquired, and the mean values were calculated and displayed by the device. The SD-OCT biometry module does not provide keratometric measurements; therefore, keratometric values from a separate device are required for intraocular lens power calculation, as with ultrasound biometry. Only examinations with an image quality score of ≥6/10, the manufacturer-defined minimum acceptable quality threshold, were included in the analysis.

Ultrasound biometry was performed using a contact A-scan biometer, equipped with a 10 MHz probe. Ultrasound velocities were set at 1532 m/s for the aqueous humor, 1641 m/s for the crystalline lens, and 1532 m/s for the vitreous humor. All measurements were performed after topical anesthesia by the same ophthalmologist with 13 years of clinical experience, who was masked to the results of the other biometry methods. Gain settings ranged from 60 to 80 depending on lens opacity. The probe was applied with minimal corneal pressure. Five consecutive measurements were obtained, and the mean value was automatically calculated by the device.

Optical biometry was performed using the SW-9000 Plus (Suowei Electronic Technology Co., Ltd., Tianjin, China), a fully automated, noncontact optical biometer based on optical low-coherence reflectometry (OLCR). The device uses an 820 nm superluminescent diode and provides AL and ACD measurements with a resolution of 0.01 mm. All examinations were performed by the same ophthalmologist using the automatic acquisition mode, which incorporates automatic alignment, focusing, and measurement acquisition. Five consecutive measurements were obtained, and their mean values were calculated by the device. Measurement quality was assessed using the integrated quality indicator, and measurements outside the green acceptance range were rejected. The device achieved measurement success rates of 100% and 80.0% in the RLE and cataract groups, respectively.

ACD and AL measurements obtained with SD-OCT biometry, optical biometry, and ultrasound biometry were evaluated separately in the RLE and cataract groups. For each group, biometric values were compared among the three methods, and interdevice agreement was assessed for ACD and AL. Measurement success was also evaluated for each method to determine its ability to provide complete biometric data under different degrees of lens opacity. A measurement was considered successful when both ACD and AL values were obtained and met the quality criteria of the corresponding device. Agreement analyses were performed using eyes with successful measurements from the methods being compared.

### Statistical Analysis

Statistical analyses were performed using IBM SPSS Statistics version 20 (IBM Corp., Armonk, NY, USA). Continuous variables are presented as mean ± standard deviation and categorical variables as number and percentage. Normality was assessed using the Shapiro–Wilk test. An ICC-based sample size calculation indicated that at least 57 eyes were required, assuming 80% statistical power and a significance level of 0.05. Within each group, ACD and AL measurements obtained with SD-OCT biometry were separately compared with those obtained with ultrasound and optical biometry using the Wilcoxon signed-rank test. Interdevice agreement was assessed separately for SD-OCT versus ultrasound biometry and SD-OCT versus optical biometry using intraclass correlation coefficients (ICCs) and Bland–Altman analysis. ICC values were classified as poor (<0.50), moderate (0.50–0.75), good (0.75–0.90), or excellent (>0.90). Bland–Altman analysis was used to determine the mean bias and 95% limits of agreement for each comparison. Bonferroni correction was applied to the two planned pairwise comparisons for each biometric parameter within each study group, and adjusted *p* values were reported. A *p* value <0.05 was considered statistically significant. The data supporting the findings of this study are available from the corresponding author upon reasonable request.

## 3. Results

This study included 207 eyes of 207 patients, with 62 eyes in the refractive lens extraction group and 145 eyes in the cataract group. Baseline demographic and clinical characteristics are presented in Table 1.

Successful biometric measurements were obtained in all eyes in the RLE group using SD-OCT biometry, ultrasound biometry, and optical biometry. In the cataract group, successful measurements were obtained in 70 eyes (48.3%) with SD-OCT biometry, 145 eyes (100%) with ultrasound biometry, and 116 eyes (80%) with optical biometry. Measurement failure rates increased with nuclear cataract severity. For LOCS III nuclear opalescence Grades 3, 4, 5, and 6, SD-OCT biometry failure rates were 4.0%, 28.3%, 85.7%, and 100%, respectively, compared with 0%, 13.2%, 25.0%, and 72.7% for optical biometry. Ultrasound biometry achieved successful measurements in all grades. Among participants with nuclear opalescence of Grade 4 or higher according to the LOCS III classification, the measurement success rate was 38.3% (46/120) for SD-OCT biometry, 75.8% (91/120) for optical biometry, and 100% (120/120) for ultrasound biometry (Table 2). For SD-OCT biometry, AL and ACD were reported together, precluding separate assessment of their measurement success rates. In contrast, optical biometry achieved an ACD measurement success rate of 100% in both groups.

In the RLE group, mean ACD measurements were 3.15 ± 0.44 mm, 3.17 ± 0.38 mm, and 3.08 ± 0.41 mm with SD-OCT biometry, ultrasound biometry, and optical biometry, respectively. Pairwise comparisons revealed no significant difference between SD-OCT biometry and ultrasound biometry (*p* = 0.170) or between SD-OCT biometry and optical biometry (*p* = 0.212). Mean AL measurements were 23.63 ± 0.98 mm, 23.52 ± 0.97 mm, and 23.56 ± 0.96 mm, respectively. AL measurements were significantly higher with SD-OCT biometry than with both ultrasound biometry (*p* < 0.001) and optical biometry (*p* = 0.003) (Table 3).

**Figure 2:**
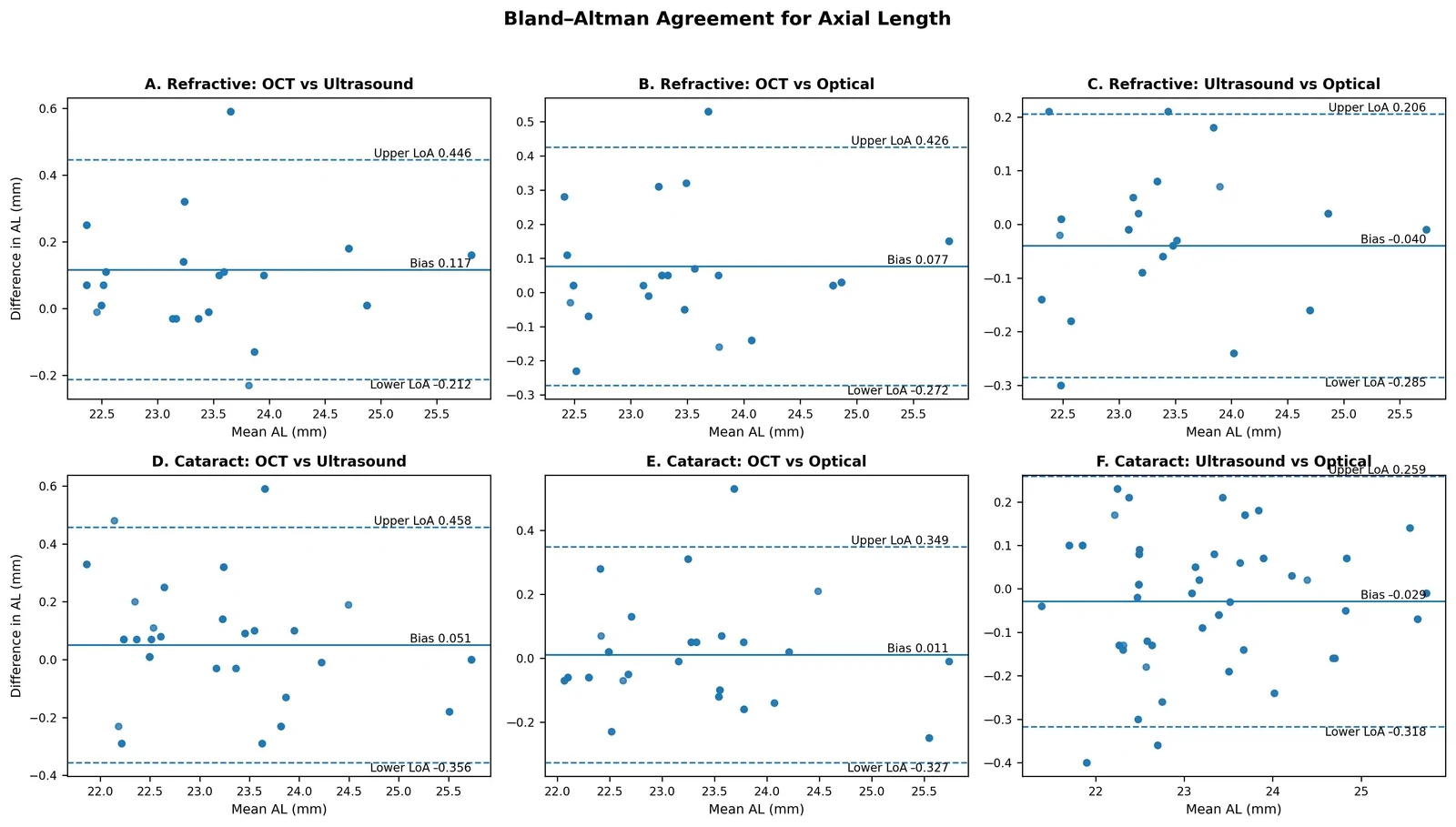
Bland–Altman plots for AL measurements. Panels (**A**–**C**) represent the RLE group: (**A**) SD–OCT versus ultrasound biometry, (**B**) SD–OCT versus optical biometry, and (**C**) ultrasound versus optical biometry. Panels (**D**–**F**) represent the cataract group: (**D**) SD–OCT versus ultrasound biometry, (**E**) SD–OCT versus optical biometry, and (**F**) ultrasound versus optical biometry. Solid horizontal lines indicate the mean bias, and dashed lines indicate the 95% limits of agreement.

**Figure 3:**
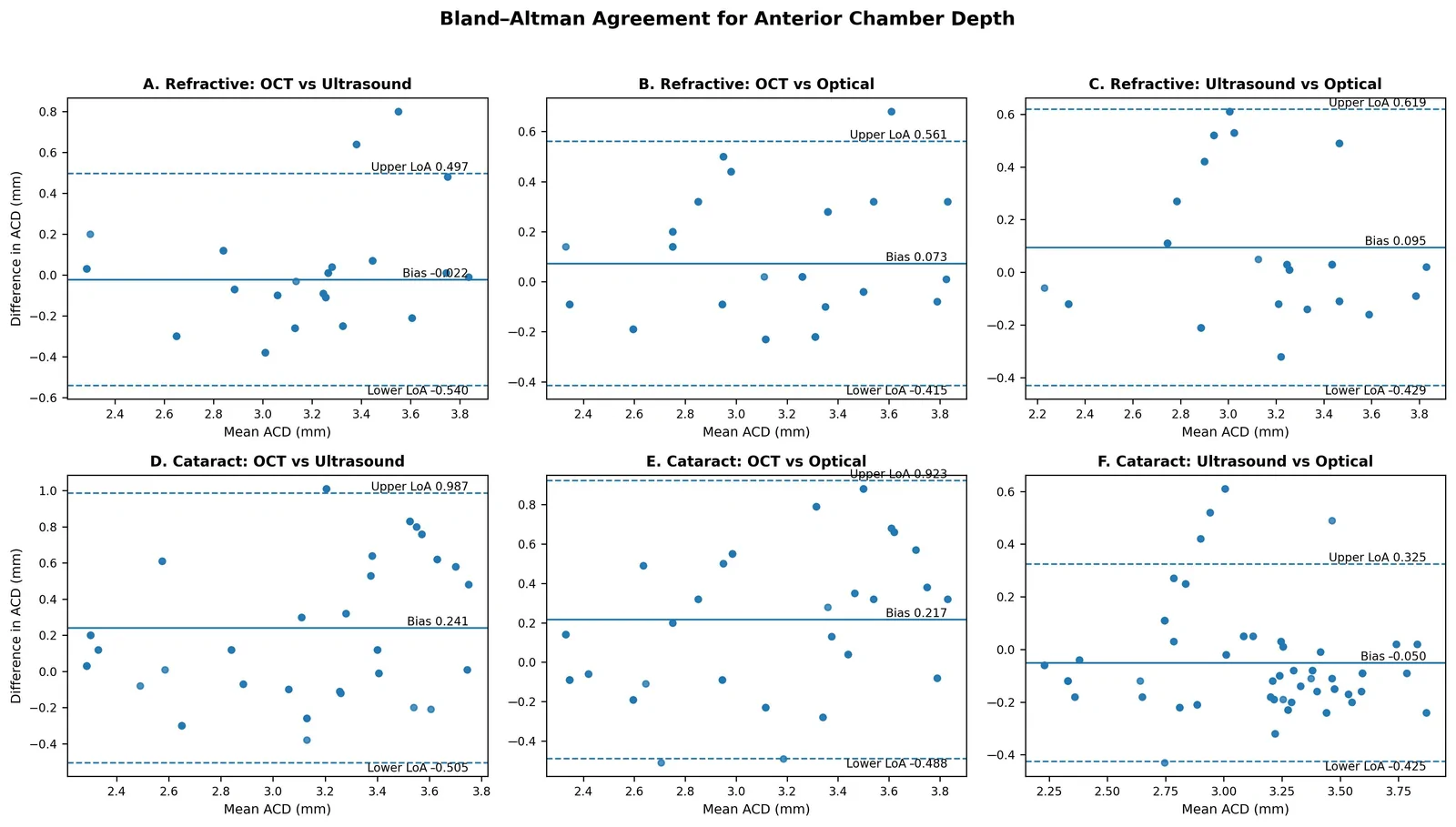
Bland–Altman plots for anterior chamber depth (ACD) measurements. Panels (**A**–**C**) represent the RLE group: (**A**) SD–OCT versus ultrasound biometry, (**B**) SD–OCT versus optical biometry, and (**C**) ultrasound versus optical biometry. Panels (**D**–**F**) represent the cataract group: (**D**) SD–OCT versus ultrasound biometry, (**E**) SD–OCT versus optical biometry, and (**F**) ultrasound versus optical biometry. Solid horizontal lines indicate the mean bias, and dashed lines indicate the 95% limits of agreement.

Pairwise agreement in the refractive lens extraction group was good for ACD (ICC = 0.799 for SD-OCT biometry vs. ultrasound biometry and ICC = 0.822 for SD-OCT biometry vs. optical biometry) and excellent for AL (ICC = 0.978 and ICC = 0.980, respectively) (Table 4). Bland–Altman analysis demonstrated small biases for AL across the device comparisons, whereas wider limits of agreement were observed for ACD (Figure 2 and Figure 3; Table 4).

In the cataract group, pairwise analyses were performed using eyes with successful measurements from both devices in each comparison (*n* = 70 for SD-OCT versus ultrasound biometry and *n* = 66 for SD-OCT versus optical biometry). For the SD-OCT versus optical biometry comparison, mean ACD measurements were 3.22 ± 0.59 mm and 3.00 ± 0.44 mm, respectively. For the SD-OCT versus ultrasound biometry comparison, mean ACD measurements were 3.23 ± 0.58 mm and 2.99 ± 0.44 mm, respectively. SD-OCT ACD measurements were significantly higher in both comparisons (*p* < 0.001). For AL, SD-OCT versus ultrasound means were 23.31 ± 1.06 mm and 23.26 ± 1.10 mm (*p* = 0.066), while SD-OCT versus optical means were 23.21 ± 0.95 mm and 23.20 ± 0.96 mm (*p* = 0.859). Neither AL comparison was statistically significant (Table 3).

Pairwise agreement in the cataract group was moderate for ACD (ICC = 0.653 for SD-OCT biometry vs. ultrasound biometry and ICC = 0.702 for SD-OCT biometry vs. optical biometry) and excellent for AL (ICC = 0.981 and ICC = 0.984, respectively) (Table 4). Bland–Altman analysis again demonstrated small AL biases, whereas ACD measurements showed larger biases and wider limits of agreement (Figure 2 and Figure 3; Table 4).

## 4. Discussion

The present study provides an independent clinical evaluation of SD-OCT biometry, a recently introduced method in which ocular biometry is carried out using a commercially available SD-OCT platform. Previous studies demonstrated high repeatability and agreement with dedicated optical biometers [8,9]. However, clinical evidence remains limited, particularly regarding its performance across different degrees of lens opacity. Ultrasound biometry provides reliable measurements even in dense cataracts because it is independent of optical media transparency. Therefore, in the present study, ultrasound biometry served as an independent comparator, particularly when only one of the two optical systems successfully obtained a measurement. By comparing SD-OCT biometry with both optical and ultrasound biometry, the present study provides a broader assessment of its measurement agreement, acquisition success, and current clinical applicability.

In the study by Sikorski et al. [8], SD-OCT biometry demonstrated remarkably close agreement with the IOL Master 700 for AL. Notably, the mean interdevice differences were exceptionally small, with the two devices approaching numerical equivalence. Such close correspondence represents a particularly high level of agreement and may not necessarily be reproduced across different clinical populations, devices, or measurement principles. In the present study, interdevice differences were somewhat larger, while overall agreement remained excellent. This may partly reflect the use of different comparator technologies. Whereas Sikorski et al. [8] compared SD-OCT biometry with an SS-OCT biometer, the present study included both dedicated optical and contact ultrasound biometry. These methods differ in their measurement principles, anatomical endpoints, assumptions regarding refractive index or sound velocity, and susceptibility to alignment or corneal compression. Small systematic differences between biometric technologies are well documented [2,3,4,11,12]. Therefore, agreement rather than numerical identity may provide a more appropriate basis for comparing measurements obtained using different biometric technologies.

AL was treated as a key biometric parameter in the present study because its accurate measurement is very important for achieving reliable refractive outcomes after refractive surgery [1,2]. The refractive impact of AL measurement differences varies with the initial axial length. For example, a 0.10 mm difference in AL measurements may correspond to approximately 0.40 D of refractive prediction difference in short eyes, compared with approximately 0.10–0.15 D in long eyes. In the RLE group, the AL measured by SD-OCT biometry was approximately 0.12 mm longer than that measured by ultrasound biometry and 0.08 mm longer than optical biometry. In cataractous eyes successfully measured with all three methods, the corresponding AL differences were substantially smaller (0.04 mm and 0.01 mm), theoretically corresponding to 0.10 to 0.12 D and 0.03 D, respectively. These estimates provide a clinical context for interpreting the observed interdevice differences; the actual refractive effect may vary according to ocular dimensions, IOL power, and the calculation formula used.

Agreement analyses provide information beyond comparisons of mean values. AL measurements demonstrated excellent ICC values, and Bland–Altman analysis showed small systematic biases between SD-OCT biometry and the other methods. However, despite these small mean biases, the 95% limits of agreement were relatively wide, indicating that larger interdevice differences may occur at the individual eye level. This distinction is important because small mean differences and excellent ICC values do not necessarily imply complete interchangeability between devices. Similar findings have been reported in comparisons among PCI, optical low-coherence reflectometry, SS-OCT, and ultrasound biometers [13,14,15]. Therefore, although the present findings support high overall consistency in AL measurements obtained with SD-OCT biometry when successful acquisition is achieved, the observed limits of agreement should be considered when interpreting measurements in individual eyes.

The clinical applicability of the current SD-OCT biometry implementation was primarily limited by measurement acquisition in cataractous eyes. Measurement success was 100% in the RLE group but decreased to 48% in the cataract group, compared with 80% for optical biometry, and declined further to 38% with SD-OCT in eyes with dense nuclear cataracts. All measurement failures in both optical methods were attributable to insufficient signal quality caused by media opacity. This finding is clinically relevant because a method intended to function as the sole biometry system in a cataract practice should provide successful measurements across a broad range of lens opacities. At its current stage, the substantially lower measurement success of SD-OCT biometry in cataractous eyes limits its applicability as a standalone biometry method.

In this study, the cataract group was restricted to eyes with nuclear cataract to provide a more homogeneous assessment of the effect of lens opacity on measurement performance. Posterior subcapsular cataracts were not included because their location may affect optical measurement performance independently of overall lens density, introducing an additional source of variability. Moreover, advanced posterior subcapsular opacities frequently prevent successful measurements with both optical systems, limiting meaningful comparison between them.

The lower measurement success observed in cataractous eyes is consistent with, although more pronounced than, previous findings. Zarei-Ghanavati et al. [9] reported successful AL acquisition in 82% of cataractous eyes with OCT biometry, compared with 91% and 97% with dedicated optical biometers. Despite differences in absolute success rates, studies demonstrate reduced acquisition success of SD-OCT biometry in the presence of cataract. Importantly, differences in the severity and type of lens opacity between study populations may substantially influence measurement success, as increasing media opacity impairs optical signal transmission and limits reliable detection of the retinal measurement endpoint. These findings indicate that lens opacity is a key determinant of measurement success and, consequently, the clinical applicability of SD-OCT biometry.

The difference in acquisition success can largely be explained by the optical characteristics and acquisition architecture of the technologies. SD-OCT biometry is implemented on a conventional SD-OCT platform rather than on a device designed specifically as an optical biometer. Successful AL measurement requires adequate transmission of the OCT signal through the ocular media and reliable detection of the relevant anatomical interfaces. Increasing lens opacity attenuates and scatters the incident and returning light, reducing signal strength and making identification of the retinal interface more difficult. Dedicated SS-OCT biometers generally use longer wavelengths and acquisition systems optimized for whole-eye imaging and provide improved penetration through opaque media [16,17,18].

The advantage of dedicated optical biometers becomes particularly relevant with increasing lens opacity. Gonzalez-Godinez et al. [15] reported an AL measurement failure rate of 68.6% with PCI compared with 21.4% with SS-OCT in dense cataracts. Modern SS-OCT biometers have similarly demonstrated substantially higher acquisition success in dense cataracts, with several studies reporting success rates approaching 100% [19,20,21]. Akman et al. [5] also reported lower measurement failure with the IOL Master 700 than with the PCI-based IOL Master 500. These technological differences likely contribute to the higher measurement success of dedicated optical biometry compared with SD-OCT biometry in cataractous eyes.

The present study highlights an important difference between measurement success and interdevice agreement. Despite the low acquisition success of SD-OCT biometry in cataractous eyes, AL measurements showed excellent agreement with the other methods when successfully obtained. Mean AL measurements obtained with SD-OCT biometry did not differ significantly from those obtained with either ultrasound or optical biometry in the cataract group, and agreement remained excellent. These findings indicate that the principal current limitation of SD-OCT biometry in cataractous eyes is its ability to obtain a measurement rather than the agreement of AL values once successful acquisition is achieved.

The potential value of SD-OCT biometry lies primarily in its multifunctionality rather than in replacing advanced dedicated biometers. OCT systems are already widely used in ophthalmic practice, and incorporating AL measurement into a commercially available SD-OCT platform could extend the functionality of a single device beyond retinal, optic nerve, and anterior segment imaging. The original description of this method (B-OCT) by Sikorski et al. similarly emphasized the potential benefit of combining ocular imaging and biometry within a single platform [8]. Such integration may offer practical and economic advantages in selected clinical settings. However, the low acquisition success in cataractous eyes currently limits its use as a standalone biometry method, and access to an alternative technique, particularly ultrasound biometry, remains necessary when optical acquisition fails. Improvements in signal penetration, acquisition algorithms, and retinal endpoint detection may substantially improve the future clinical applicability of SD-OCT biometry.

Accurate ACD measurement is clinically important because it contributes to effective lens position estimation, IOL power calculation, appropriate lens selection, and anticipation of potential intraoperative challenges, ultimately influencing surgical planning and postoperative refractive outcomes. The wider Bland–Altman limits of agreement observed for ACD indicate greater interdevice variability, suggesting that ACD measurements obtained using these devices should not be considered directly interchangeable in individual patients. ACD is more dependent on device-specific measurement principles and the identification of anatomical interfaces, particularly the corneal and anterior lens surfaces. Differences in imaging technology, wavelength, scan geometry, image resolution, and automated boundary detection can therefore produce systematic differences among devices. Recent studies comparing SS-OCT, OLCR, and Scheimpflug-based systems have also demonstrated device-dependent differences in ACD measurements [22,23,24]. Contact ultrasound introduces additional variability because probe positioning and corneal compression can affect the measured chamber depth. These technical differences explain why ACD measurements may be less interchangeable among biometric devices than AL measurements.

The present study has several strengths. It provides an independent evaluation of a practical biometry method for which clinical evidence remains limited. SD-OCT biometry was compared with both optical and ultrasound biometry, allowing assessment against two established methods based on different measurement principles. Separate evaluation of eyes with minimal lens opacity and cataract also allowed measurement success to be assessed under different optical conditions. Interdevice agreement was evaluated using both ICC and Bland–Altman analyses.

The present study has several limitations. It was conducted at a single center and included one optical biometer and one ultrasound biometer, which may limit the generalizability of the findings to other biometric systems. Although minimal corneal pressure was applied during contact ultrasound biometry, corneal compression was not objectively quantified. In the cataract group, agreement analyses included only eyes with successful measurements. In addition, this study focused on biometric measurements rather than on IOL power calculations or postoperative refractive outcomes. Therefore, although the observed AL differences can be interpreted in the context of their estimated effect on IOL power prediction, their actual refractive consequences require further evaluation.

## 5. Conclusions

SD-OCT biometry represents a practical approach that extends ocular biometry to a commercially available SD-OCT platform. The method demonstrated excellent agreement with established techniques for AL when measurements were successfully obtained. Its principal current limitation is the markedly lower measurement success in cataractous eyes than in dedicated optical and ultrasound biometry, limiting its use as a standalone biometry method in cataract practice. Nevertheless, the high agreement of successfully obtained AL measurements and the integration of biometry into a multifunctional SD-OCT platform support further development of this technology. Advances in signal penetration and acquisition through lens opacity may improve the clinical applicability of SD-OCT biometry.

## Figures and Tables

**Table 1:** Demographic and clinical characteristics of the study groups.

Variable	Refractive Lens Extraction Group (*n* = 62)	Cataract Group (*n* = 145)
Age (years), mean ± SD	48.66 ± 7.09	66.66 ± 8.47
Male, *n* (%)	42 (67.7)	66 (45.5)
Female, *n* (%)	20 (32.3)	79 (54.5)
BCVA (logMAR), mean ± SD	0.03 ± 0.08	0.68 ± 0.16
AL (mm), mean ± SD (ultrasound)	23.52 ± 0.97	23.38 ± 1.23
ACD (mm), mean ± SD (ultrasound)	3.17 ± 0.38	3.12 ± 0.39
LOCS III NO grade, mean ± SD	1.03 ± 0.72	4.37 ± 0.86
Dense cataract (NO ≥ 4), *n* (%)	0 (0.0)	120 (82.8)
LOCS III NO grade 0–2, *n* (%)	60 (96.8)	0 (0.0)
LOCS III NO grade 3, *n* (%)	2 (3.2)	25 (17.2)
LOCS III NO grade 4, *n* (%)	0 (0.0)	53 (36.6)
LOCS III NO grade 5, *n* (%)	0 (0.0)	56 (38.6)
LOCS III NO grade 6, *n* (%)	0 (0.0)	11 (7.6)

Values are presented as mean ± standard deviation (SD) or *n* (%). Baseline AL and ACD values were obtained using ultrasound biometry. LOCS III, Lens Opacities Classification System III; NO, nuclear opalescence. BCVA, best-corrected visual acuity; logMAR, logarithm of the minimum angle of resolution; AL, axial length; ACD, anterior chamber depth; RLE, refractive lens extraction.

**Table 2:** Measurement success rates of the three biometry methods.

Method	Refractive Lens Extraction Group (*n* = 62)	Cataract Group (*n* = 145)	Nuclear Opalescence ≥ Grade 4 (*n* = 120)
SD-OCT biometry	62/62 (100.0%)	70/145 (48.3%)	46/120 (38.3%)
Ultrasound biometry	62/62 (100.0%)	145/145 (100.0%)	120/120 (100.0%)
Optical biometry	62/62 (100.0%)	116/145 (80.0%)	91/120 (75.8%)

Values are presented as successful measurements/total number of eyes (%). Nuclear opalescence ≥ Grade 4 represents the dense nuclear cataract subgroup. Success denotes acquisition of both AL and ACD for SD-OCT and ultrasound biometry; for optical biometry, the rates shown correspond to AL acquisition, while ACD was acquired in all eyes.

**Table 3:** Pairwise comparison of ACD and AL measurements in the cataract and RLE groups.

Group	Parameter	Comparison	*n*	SD-OCT Biometry Mean ± SD	Comparator Mean ± SD	*p* Value
RLE	ACD (mm)	SD-OCT biometry vs. ultrasound biometry	62	3.15 ± 0.44	3.17 ± 0.38	0.170
RLE	ACD (mm)	SD-OCT biometry vs. optical biometry	62	3.15 ± 0.44	3.08 ± 0.41	0.212
RLE	AL (mm)	SD-OCT biometry vs. ultrasound biometry	62	23.63 ± 0.98	23.52 ± 0.97	<0.001
RLE	AL (mm)	SD-OCT biometry vs. optical biometry	62	23.63 ± 0.98	23.56 ± 0.96	0.003
Cataract	ACD (mm)	SD-OCT biometry vs. ultrasound biometry	70	3.23 ± 0.58	2.99 ± 0.44	<0.001
Cataract	ACD (mm)	SD-OCT biometry vs. optical biometry	66	3.22 ± 0.59	3.00 ± 0.44	<0.001
Cataract	AL (mm)	SD-OCT biometry vs. ultrasound biometry	70	23.31 ± 1.06	23.26 ± 1.10	0.066
Cataract	AL (mm)	SD-OCT biometry vs. optical biometry	66	23.21 ± 0.95	23.20 ± 0.96	0.859

Data are presented as mean ± standard deviation (SD). Pairwise comparisons were performed using the Wilcoxon signed-rank test; Bonferroni-adjusted *p* values are reported. The sample size (*n*) represents eyes with successful measurements from both devices in each comparison. ACD, anterior chamber depth; AL, axial length; RLE, refractive lens extraction; SD-OCT, spectral-domain optical coherence tomography.

**Table 4:** Pairwise interdevice agreement and Bland–Altman analysis of biometric measurements.

Group	Parameter	Comparison	*n*	ICC (95% CI)	Bias (mm)	Lower 95% LoA	Upper 95% LoA
RLE	AL	SD-OCT biometry vs. Ultrasound	62	0.978 (0.958–0.989)	0.117	−0.212	0.446
RLE	AL	SD-OCT biometry vs. Optical	62	0.980 (0.963–0.990)	0.077	−0.272	0.426
RLE	ACD	SD-OCT biometry vs. Ultrasound	62	0.799 (0.654–0.891)	−0.022	−0.540	0.497
RLE	ACD	SD-OCT biometry vs. Optical	62	0.822 (0.726–0.888)	0.073	−0.415	0.561
Cataract	AL	SD-OCT biometry vs. Ultrasound	70	0.981 (0.966–0.989)	0.051	−0.356	0.458
Cataract	AL	SD-OCT biometry vs. Optical	66	0.984 (0.970–0.992)	0.011	−0.327	0.349
Cataract	ACD	SD-OCT biometry vs. Ultrasound	70	0.653 (0.515–0.754)	0.241	−0.505	0.987
Cataract	ACD	SD-OCT biometry vs. Optical	66	0.702 (0.591–0.787)	0.217	−0.488	0.923

ICC, pairwise absolute-agreement intraclass correlation coefficient; CI, confidence interval (bootstrap 95% CI); bias, SD-OCT biometry minus the comparator; LoA, limit of agreement (mean bias ± 1.96 SD of paired differences). Only eyes with successful paired measurements were included. ACD, anterior chamber depth; AL, axial length; RLE, refractive lens extraction; SD, standard deviation.

## Data Availability

The data supporting the findings of this study are available from the corresponding author upon reasonable request.
